# New human hepatocellular carcinoma (HCC) cell line with highly metastatic potential (MHCC97) and its expressions of the factors associated with metastasis

**DOI:** 10.1038/sj.bjc.6690769

**Published:** 1999-11

**Authors:** J Tian, Z Y Tang, S L Ye, Y K Liu, Z Y Lin, J Chen, Q Xue

**Affiliations:** Liver Cancer Institute and Zhong-Shan Hospital, Shanghai Medical University, Shanghai 200032, Peoples Republic of China

**Keywords:** hepatocellular carcinoma, establishment, cell culture, cell line, metastasis, metastasis-associated factors

## Abstract

A new human hepatocellular carcinoma (HCC) cell line with a highly metastatic potential was established from subcutaneous xenograft of a metastatic model of human HCC in nude mice (LCI-D20) by means of alternating cell culture in vitro and growth in nude mice. The line, designated MHCC97, has been cultivated for 18 months and subcultured for more than 90 passages. The line was showed to be of human origin by karyotype analysis. The cells were either grown as compact colonies (in clusters) or as a monolayered sheet with about 31 h of population-doubling time, exhibited typical malignant epithelial in morphology and were positive for α-fetoprotein (AFP). Flow cytometric analysis of the cell DNA content showed an aneuploid pattern, and its index was 1.5 as compared to that of normal human peripheral blood lymphocytes. Karyotypic analyses of G- and C-banding techniques revealed that all cells presented chromosome abnormalities in number and structure. The number of cell line MHCC97 chromosome ranged from 59 to 65 with a modal number of 60 and 61. At least two common chromosome markers, i(1q) and der(4)t(4;?)(4pter→q35::?), were present in all cells, and deletion of Y chromosome also occurred in all cells. The subcutaneous and intrahepatic xenografts were formed and metastatic lesions in lungs were found after the cells were inoculated into nude mice. The rate of metastasis to lungs was 100% using orthotopic inoculation. Reverse transcription polymerase chain reaction products revealed positive expressions of integrin α_5_ and β_1_, urokinase type plasminogen activator receptor (uPAR), vascular endothelial growth factor and nm23-H1 mRNAs of cell line MHCC97. Immunostaining of c-Met, uPAR showed strongly positive in both subcutaneous xenografts and lung metastatic lesions; while positive in xenografts and negative in metastatic lesions for integrin α_5_, β_1_. E-cadherin and P53 was not expressed either in xenograft or in the metastatic lesions. PCR products of HBsAg and HBxAg were both positive. The cell line MHCC97 still retained some characteristic features of original tumour. Establishment of cell line MHCC97 should be beneficial to the studies of HCC metastatic mechanisms. © 1999 Cancer Research Campaign


*British Journal of Cancer* (1999) **81**(5), 814–821
© 1999 Cancer Research Campaign
Article no. bjoc.1999.0769

### New human hepatocellular carcinoma (HCC) cell line with highly metastatic potential (MHCC97) and its expressions of the factors associated with metastasis

**J Tian, ZY Tang, SL Ye, YK Liu, ZY Lin, J Chen and Q Xue**

Liver Cancer Institute and Zhong-Shan Hospital, Shanghai Medical University, Shanghai 200032, Peoples Republic of China

**Summary** A new human hepatocellular carcinoma (HCC) cell line with a highly metastatic potential was established from subcutaneous xenograft of a metastatic model of human HCC in nude mice (LCI-D20) by means of alternating cell culture in vitro and growth in nude mice. The line, designated MHCC97, has been cultivated for 18 months and subcultured for more than 90 passages. The line was showed to be of human origin by karyotype analysis. The cells were either grown as compact colonies (in clusters) or as a monolayered sheet with about 31 h of population-doubling time, exhibited typical malignant epithelial in morphology and were positive for α-fetoprotein (AFP). Flow cytometric analysis of the cell DNA content showed an aneuploid pattern, and its index was 1.5 as compared to that of normal human peripheral blood lymphocytes. Karyotypic analyses of G- and C-banding techniques revealed that all cells presented chromosome abnormalities in number and structure. The number of cell line MHCC97 chromosome ranged from 59 to 65 with a modal number of 60 and 61. At least two common chromosome markers, i(1q) and der(4)t(4;?)(4pter→q35::?), were present in all cells, and deletion of Y chromosome also occurred in all cells. The subcutaneous and intrahepatic xenografts were formed and metastatic lesions in lungs were found after the cells were inoculated into nude mice. The rate of metastasis to lungs was 100% using orthotopic inoculation. Reverse transcription polymerase chain reaction products revealed positive expressions of integrin $\alpha_5$ and $\beta_1$, urokinase type plasminogen activator receptor (uPAR), vascular endothelial growth factor and nm23-H1 mRNAs of cell line MHCC97. Immunostaining of c-Met, uPAR showed strongly positive in both subcutaneous xenografts and lung metastatic lesions; while positive in xenografts and negative in metastatic lesions for integrin $\alpha_5$, $\beta_1$. E-cadherin and P53 was not expressed either in xenograft or in the metastatic lesions. PCR products of HBsAg and HBxAg were both positive. The cell line MHCC97 still retained some characteristic features of original tumour. Establishment of cell line MHCC97 should be beneficial to the studies of HCC metastatic mechanisms. © 1999 Cancer Research Campaign

**Keywords**: hepatocellular carcinoma; establishment; cell culture; cell line; metastasis; metastasis-associated factors

Human hepatocellular carcinoma (HCC) is one of the most common types of tumour, especially in Asia and Africa (Anderson et al, 1992; Okuda, 1992; Parkin et al, 1993). HCC is characterized by extensive carcinoma cell infiltration and metastasis. Although advances have been made in liver cancer therapies, most cancer deaths still result from metastatic disease, so metastasis has been the greatest obstacle to successful tumour treatment. The poor understanding of the molecular mechanisms responsible for the disseminated metastasis, which would be partly due to the lack of ideal samples and models for study, has hindered the development of effective antimetastatic therapies. Although several reports of the establishment of HCC cell lines were available (Chen, 1963; Alexander et al, 1976; Dong et al, 1980), none have demonstrated prominent metastatic potential. The human cell line with a metastatic potential from primary tumour or metastatic lesions would permit metastatic mechanisms to be studied further. However, the cancer cells from the surgical samples or metastatic lesions can only be maintained in primary culture for a short time before cellular senescence occurs. The aim of this study was to establish a human HCC cell line with a metastatic potential. We successfully established a new HCC cell line with a highly metastatic potential derived from subcutaneous xenograft of a metastatic model of human HCC in nude mice (LCI-D20) (Sun et al, 1996) by improving the conventional method of establishing a cell line – alternating cell culture in vivo and growth in nude mice. In this paper the processes of establishing a new human HCC cell line MHCC97 and some characteristics, including observation of some factors associated with metastasis, are reported.

*Received 24 November 1998*
*Revised 24 March 1999*
*Accepted 10 May 1999*

*Correspondence to:* J Tian, Department of Physiology and Pharmacology, School of Medicine, Loma Lind University, Loma Linda, CA 92350;
E-mail: jtian@som.llu.edu

### MATERIALS AND METHODS

### Specimens and in vitro culture

Seventy-eight primary cultures were taken from subcutaneous or intrahepatic xenografts of LCI-D20 nude mouse models. The model was developed by orthotopic inoculation of an intact tumour tissue of an intrahepatic disseminated lesion from a 39-year-old Chinese male patient with HCC (Zhong-Shan Hospital, Shanghai Medical University). Abnormal alpha-fetoprotein (AFP) and HBsAg were found in serum from this patient. The LCI-D20 model represents 100% metastases to the lungs, lymph nodes and intrahepatic spreading (Sun, 1996).

These xenografts were removed and used for primary culture in vitro by different culture methods (tissue fragments or single-cell suspension). Different culture mediums (RPMI-1640, Dulbecco's modified Eagle's medium (DMEM) or DMEM/RPMI-1640; Gibco-BRL) with fetal calf serum (FCS; Gibco-BRL), newly born serum (NBS; Shanghai Institute of Cell Biology, Academiae Sinica) or human blood group AB serum (purchased from the Blood Center, Shanghai) were employed. Briefly, about ten 1–2 $mm^2$ fragments were transferred to a 25-$cm^2$ plastic culture flask (Nunclon™, Denmark) with RPMI-1640 or DMEM containing 10% of one kind of serum above. The medium containing single-cell suspension obtained by tearing xenograft in serum-free DMEM was transferred to a 10-ml centrifuge tube and allowed to stand undisturbed for about 5–10 min until the small pieces of tissue and clusters of cell settled at the bottom of the tube. The suspension with dispersed cells was aspirated into another tube and centrifuged slightly. The pellet was washed once in medium by light centrifugation. Cells were resuspended with DMEM supplemented with 10% heated-inactivated human blood group AB serum without antibiotics and seeded into 25-$cm^2$ plastic culture flasks (Nunclon™, Denmark) at 37°C in a humidified atmosphere with 5% carbon dioxide. Twenty-four hours after seeding, the cultures were washed in DMEM and fed with fresh complete medium; the medium was routinely changed every 2–3 days. At confluence the cells were subcultured by exposure to trypsin–versene (0.25% trypsin with 0.02% EDTA) in a calcium ion ($Ca^{2+}$) and magnesium ion ($Mg^{2+}$)-free Hank's solution. In order to ensure tumour cells for long-term culture in vitro, we developed a new culture method by modifying conventional method of establishing cell line. Briefly, after cultured for 1.5 months or subcultured 5–7 passages before cellular senescence occurred, the cells ($\geq 10^6$) were returned to the subcutis of nude mice (BALB/c) until tumour nodules formed and then were re-treated as above. This procedure was repeated 5–6 times. The cancer cells treated in this way could grow steadily and be subcultured successfully in vitro. The 33258 Hoechst stain was routinely employed for *Mycoplasma* testing.

### Determination of doubling time

The cells of passage 40 were studied to estimate the population-doubling time. The initial cell number was $4 \times 10^4$ cells $well^{-1}$ in triplicate in 24-well plate (Nunclon, USA). A cell count was taken every 2 days in three wells, and the growth medium was changed every 2 days. The number of viable cells was determined in a haemocytometer every 48 h for 20 days by trypan blue staining. The doubling time of the cell line MHCC97 was calculated during the exponential growth phases.

### Morphology and ultrastructure of the MHCC97 cell line

Cells in flasks were photographed directly without stain under the phase-microscope. For electron microscopic analysis, the monolayer cells were scraped from flasks and pelleted. The pellet was fixed with 2.5% glutaraldehyde in 0.1 mol phosphate-buttered saline (PBS), pH 7.2, washed in 0.1 mol PBS, and then post-fixed in 2% osmium tetraoxide after dehydration in ethanol; then the samples were embedded in Epon resin. Ultrathin sections were stained with uranyl acetate and lead citrate, and examined under a JEOL 1200EX (JEOL, Tokyo, Japan) electron microscope.

### Histology of xenografts

Routine histology was carried out in order to compare morphology of original xenografts with that of tumour xenografts developed after re-inoculation of cultured MHCC97 cells. The subcutaneous and intrahepatic xenografts were fixed in 10% neutral formalin, processed routinely, and paraffin sections stained with haematoxylin and eosin (H&E).

### Measurement of DNA content by flow cytometry

DNA content of nuclei from cultured cells at the 30th passage was analysed by flow cytometry. In brief, cultured cells were trypsinized and the resulting suspension transferred to a 10-ml centrifuge tube for centrifugation, resuspended in 10 ml PBS and pelleted once before being resuspended in citrate buffer. A total of $1 \times 10^6$ cells was treated with 1.8 ml of trypsin, and then propidium iodide (0.25 mg $ml^{-1}$), ribonuclease (5 mg $ml^{-1}$) and Triton X-100 (1%) were added for more than 15 min prior to analysis. Cells were analysed using a FACScan machine (Becton-Dickinson). The DNA index was calculated relative to normal adult peripheral blood lymphocytes.

### Cytogenetic analysis

Chromosome preparation and G-banding were performed as Seabright (1971) described, but slightly modified. Briefly, colcemid was added to cell cultures at the 35th passage to a final concentration of 0.25 μg $ml^{-1}$ for 10–15 h. The cells were then trypsinized and centrifuged at 1200 rpm for 12 min. The hypotonic medium used was 0.075 mol potassium chloride for 20 min at 37°C, and then the fixative (methanol:glacial acetic acid, 3:1) was changed three times before slides were made. Some of the slides were aged at 60°C for 8–10 h for G-banding. The aged slides were trypsinized for 3.5 min, rinsed in water and stained with Giemsa, rinsed again, air dried and examined under microscope. The rest for C-banding was soaked in 0.2 N hydrochloric acid for 1 h, and then incubated in 5% $Ba(OH)_2$ at 56°C for 10 min and rinsed in water. After that, the slides were re-incubated with 2 × SSC (standard saline citrate) at 67°C for 1–1.5 h, rinsed again and stained with Giemsa.

### Tumorigenicity and metastasis assays in nude mice

Cultured cells at the 5th, 15th, 20th, and 35th passages were washed with $Ca^{2+}$ and $Mg^{2+}$-free Hank's solution, and 0.1–0.2 ml of a cell suspension ($> 1 \times 10^6$) was injected into the livers, subcutises or eyes of 16 weanling athymic nude mice (BALB/c *nu/nu*) respectively for tumorigenicity and spontaneous metastasis assay. The mice housed in microisolator cages under specific pathogen-free conditions, and were fed autoclaved pellets. Tumour formation was monitored every 2 days before sacrificed. The developed subcutaneous xenografts were then re-inoculated into mouse livers for spontaneous metastasis assay or eyes for tumorigenicity. All animals inoculated intrahepatically were sacrificed for metastatic assay when they appeared distressed after the initial injection of cells. For each assay, major organs (lung, liver, spleen, kidney, stomach, colon, brain and lymph nodes) were examined for the presence of metastases. Above organs were removed after inoculation for 35 days and fixed in 10% neutral formalin for paraffin-embedded sections and H&E staining. The metastatic focuses were evaluated under light microscope.

© 1999 Cancer Research Campaign

**Table 1** Oligonucleotide primer sequences used for cDNA amplification

| Factor | Primer | Primer sequence | Annealing (°C) | Product (bp) |
|---|---|---|---|---|
| Integrin α5 | Sense | 5′-ACCAAGGCCCCAGCTCCATTAG-3′ | 57 | 357 |
| | Antisense | 5′-GCCTCACACTGCAGGCTAAATG-3′ | | |
| Integrin β1 | Sense | 5′-AACTTGATCCCTAAGTCAGCAGTAG-3′ | 57 | 1200 |
| | Antisense | 5′-ATCAGCAGTAATGCAAGGCC-3′ | | |
| uPAR | Sense | 5′-GCTTAGAGAAGACGTGCAGGGA-3′ | 55 | 454 |
| | Antisense | 5′-TTCACCTTCCTGGATCCAGT-3′ | | |
| VEGF | Sense | 5′-TTGCTTGCTCTACCTCCAC-3′ | 55 | 490 |
| | Antisense | 5′-AATGCTTTCTCCGCTCTG-3′ | | |
| nm23-H1 | Sense | 5′-GCAGCCGGAGTTCAAACCTAA-3′ | 61 | 585 |
| | Antisense | 5′-GCTGGGAGGAAGCATTTTAATC-3′ | | |

### Immunohistochemical procedure

The intrahepatic xenografts developed and lungs from the nude mice inoculated with MHCC97 cells were removed and fixed in 10% neutral formalin. The paraffin-embedded sections and the cells grown on coverslips were stained by the avidin–biotin–peroxidase complex (ABC) method.

Briefly, after deparaffinizing and hydrating, these tissue sections and cells on coverslips were first soaked in methanol containing 0.6% hydrogen peroxide for 30 min to block endogenous peroxidase activity. They were then treated with 10% normal blocking serum at 37°C for 30 min to suppress non-specific stain in humidified chamber. Suction was used to remove reagents after each step, but the drying of specimens between two steps was avoided. After that, they were washed in PBS, and then incubated with individual primary antibody for 1 h at 37°C and overnight at 4°C. Then sections were re-incubated with biotinylated second-step antibodies for 1 h at 37°C and avidin–biotin enzyme reagent for 30 min respectively. Finally, sections were rinsed in 0.5% Triton X-100/PBS prior to chromogenesis with a 3,3′-diamobenzidine tetrahydrochloride and hydrogen peroxide mixture. Haematoxylin was used as a counterstain. Primary antibodies were used as follows: rabbit anti-human AFP polyclonal antibody (Dako, Denmark), murine anti-human integrin $\alpha_5$ and $\beta_1$ subunit monoclonal anti-serum (from the Department of Biochemistry, Shanghai Medical University), goat anti-human E-cadherin polyclonal antibody (Santa Cruz Biotechnology, Inc.) and rabbit anti-human uPAR (from the Department of Molecular Biology, Shanghai Medical University) and c-Met (Santa Cruz Biotechnology, Inc.) polyclonal antibody and P53 respectively. Negative controls were included in each test by substitution normal animal serum for primary antibodies.

### PCR analysis of HBV-DNA

Polymerase chain reaction (PCR) analyses were performed with HBsAg, HBcAg and HBxAg primers.

### *DNA isolation*

A total of $1 \times 10^6$ cultured cells were thoroughly homogenated in 400 μl DNA denature solution, following incubated in 100 μl 10 × SDS, 20 μl RNAase, 20 μl proteinase K at 37°C for 24 h. The DNA was extracted with phenol–chloroform. Extracted DNA was precipitated with ethanol, and resuspended in distilled water.

### *PCR procedure*

The DNA template of HBsAg, HBcAg and HBxAg primers was PCR-amplified by adding 10 μl of DNA to 50 μl final volume PCR mixture containing 3 μl of 25 mmol potassium chloride, 5 μl of 10 × amplification buffer, 1 μl of 20 mmol dNTP, 2 μl of 50 pmol HBsAg, HBcAg and HBxAg primers, and 2 units of *Taq* DNA polymerase (Promega). The complete denaturation of the DNA template was carried out at 94°C for 4 min prior to the start of PCR reaction. Thirty-five cycles were performed at 94°C for 1 min, 55°C for 1 min and 72°C for 1 min. The samples were re-incubated at 72°C for 7 min after cycles finished. The PCR products were separated by electrophoresis on a 1.2% agarose-TAE (Tris acetate buffer) and stained with ethidium bromide.

### Reverse transcription (PCR)

Total cellular RNA prepared from $1 \times 10^6$ cells using Qiagen RNeasy Mini Kit (QIAGEN, Germany). The procedure was performed according to the protocol provided by the manufacturer. Total RNA (5 μg) extracted from cell line MHCC97 was used as a template for synthesis of oligo$(dT)_{15}$-primed double-stranded cDNA using SuperScript™II RNAase $H^-$ Reverse Transcriptase (Life Technologies, Inc.). The reaction was terminated by heating at 94°C for 4 min to inactivate reverse transcription (RT) prior to the start of PCR and then 35 cycles were performed at 94°C for 1 min, annealing temperature (shown in Table 1) for 1 min, and primer extension at 72°C for 1 min. The samples were re-incubated at 72°C for 7 min after the cycles finished. The amplified products were separated by electrophoresis on a 1.2% agarose gel in Tris acetate buffer and stained using 1 μg $ml^{-1}$ ethidium bromide.

### RESULTS

### Establishment of a cell line

The 78 cultures derived from subcutaneous or intrahepatic xenografts of LCI-D20 model were used in an attempt to establish a metastatic cell line of human HCC. At last, only one, which derived from subcutaneous xenograft and was incubated in DMEM with high glucose and 10% human blood group AB serum, could be maintained and subcultured in vitro by a modified culture method of establishing a cell line – alternating cell culture in vitro and growth in nude mice. At the time of this report, the line had continuously grown for 18 months in vitro and discontinuously for more than 2 years, including the period of growth in nude mice.

 *© 1999 Cancer Research Campaign*

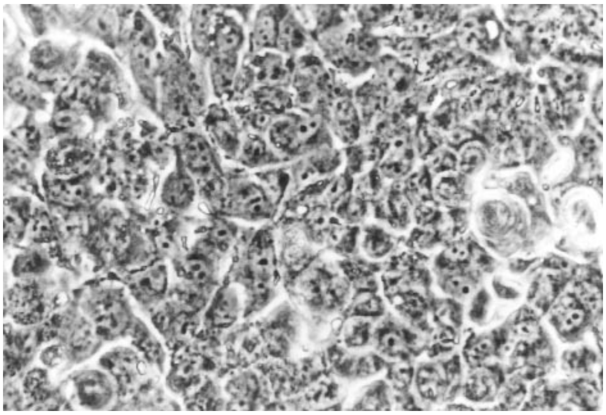

**Figure 1** Microphotograph of the cultural cell line MHCC97 (passage 35)

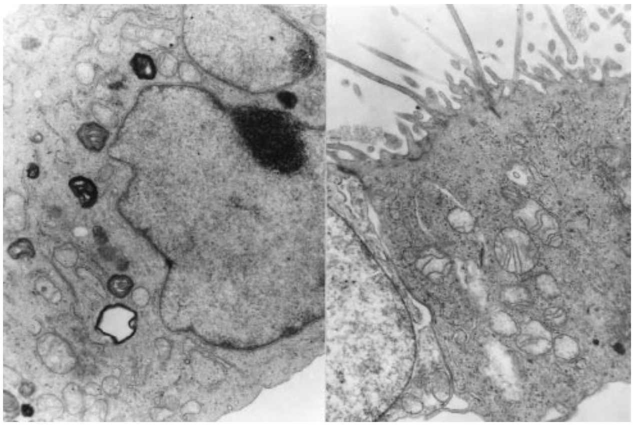

**Figure 2** Electron microphotographs of MHCC97 cells displaying irregular nuclei with deeply indented nuclear membrane, myelinoid bodies, swollen mitochondria in cytoplasm and abundant microvilli on cell surface (×7500)

### Morphology and growth pattern of MHCC97 cell line

During the first 3 weeks of the primary culture, proliferating tumour cells formed small colonies, some of which were surrounded by a fair number of fibroblasts. However, these fibroblasts gradually faded as subcultures were carried out. The cell line MHCC97 was either grown in clusters with contact inhibition loss or as monolayer polygon with clear boundaries (Figure 1). The cells were characterized by an epithelial-like shape with prominent nuclei. Ultrastructurally, most individual cells revealed large ratio of prominent irregular nuclei with deeply identified nuclear membrane relative to cytoplasm. More euchromatin and myelinoid bodies, swollen mitochondria, granular glycogen were frequently visible in MHCC97 cells. Many and long microvilli on the cell surface but few junctions were seen (Figure 2). The line grew free of *Mycoplasma* contamination and showed doubling time of 31 h.

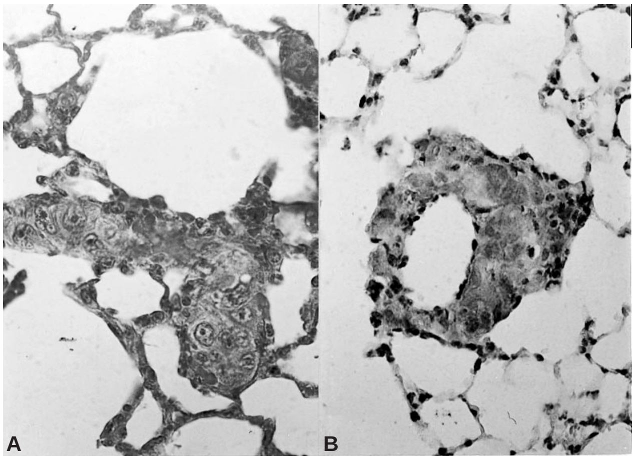

**Figure 3** Microphotographs of pulmonary metastatic lesions from nude mice with MHCC97 cell inoculations with H&E stain (A) and AFP immunostain (B) (×400). Scale bar = 25 μm

### Histology of xenografts

MHCC97 cells were implanted subcutaneously and intrahepatically in nude mice. The resultant tumours closely resembled the original xenograft maintained in nude mice in histology. The xenograft tissues were characterized by a typical pathology of hepatocellular carcinoma. The tumour cells arranged in a nest with few mesenchymes. Most of the tumour cells had large multinuclei with the nuclear membrane showing deep indentation. The multi–nucleated giant cells were often seen. Pathologically, the cancer cell of metastatic lesions in lungs were also demonstrated to have a characterization of HCC (Figure 3).

### Profile of DNA content

Analysis of DNA content from the cell line MHCC97 by flow cytometry indicated that the samples contained a major aneuploid population, with approximately 1.5 times more cells compared to that of normal human peripheral blood lymphocytes.

### Karyotype analyses of cell line MHCC97

Karyotype analyses revealed only human chromosomes with gross aneuploidy. G-banding technique showed the presence of chromosome abnormalities in number and structure in all the cells examined. The number of cell line MHCC97 chromosome ranged from 59 to 65 with a modal distribution at 60 and 61. The monosomies 13, 14 and 15 also appeared in all the cells. At least two distinctive marker chromosomes, i(1q) and der(4)t(4;?)(4pter→q35::?), were invariably present in all the cells (Figure 4), and the intact Y chromosome was absent in all the cells by G- and C-banding analyses. However, a narrow, deeply stained band inserted in the long arm of chromosome group B was noted in a few cells in C-banding karyotypes. The positive results of PCR-amplified product for DYZ-1 primer of Y chromosome may indicate that this inserted band derived from Yqh. In addition, other abnormalities, such as trisomies 5,6,11, polysomies (≥ tetrasomies) 7,12 and rearrangements of unidentified chromosomes were present in most of the cells examined (Tables 2 and 3).

### Tumorigenicity and metastatic potential in nude mice

All animals developed solid tumours at subcutis, liver and eye, with the dispersed cells or intact tissue inoculation. Tumorigenicity rate was 100%. The tumour nodules usually became visible or palpable after 15 days, and vessels were

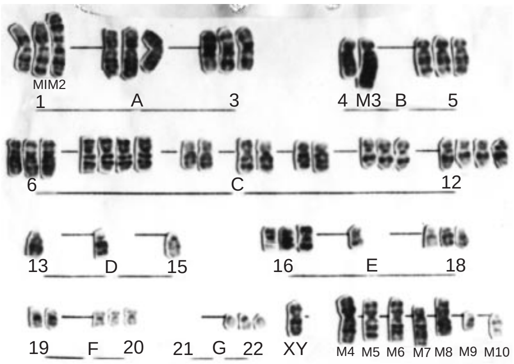

**Figure 4** G-branded karyotype of MHCC97 cell line (passage 35)

frequently induced to develop at inoculated sites. These resultant tumours grew invasively in surrounding tissues, which resembled that of the original xenograft. All autopsies, covering the lung, liver, kidney, spleen, brain, stomach and bowel, were examined histologically for metastasis assay. The resultant metastatic lesions were only found in the liver and lungs. The metastatic rate was 100% with intrahepatic inoculation. There were no prominent, enlarged lymph nodes found. Strongly positive stain for AFP was seen in both xenograft and metastatic lesions (Figure 3).

### Expression of integrin $\alpha_5$ and $\beta_1$, uPAR, VEGF and nm23-H1 mRNAs

RT-PCR was performed on total RNA extracted from cell line MHCC97 for mRNA expressions of factors associated with metastasis, such as integrin $\alpha_5$, $\beta_1$ subunits as the receptors for fibronection, urokinase type plasminogen activator receptor (uPAR) indirectely responsible for the degradation of extracellular matrix (ECM) and VEGF involved in the development of tumour vessels. All RT-PCR products of the above factors were positive for MHCC97 cells.

### Expression of integrin $\alpha_5$ and $\beta_1$, uPAR and c-Met proteins

Immunostaining patterns varied with the antibodies employed. The strongly positive stains for c-Met and uPAR confined to the cytoplasm and membrane were present in all cancer cells and xenografts, pulmonary metastatic lesions from treated nude mice. Positive results were obtained in some cancer cells of xenografts, but negative in metastatic lesions for $\alpha_5$, $\beta_1$. E-cadherin was not expressed either in xenografts or in metastatic lesions. In contrast, no positive stain was detected with normal serum (Figure 5).

### DISCUSSION

Hepatocellular carcinoma is the most frequent malignancy in China. Infection of viral hepatitis B remained one of the major backgrounds of HCC for Chinese patients. The patients often die from metastasis and recurrence of HCC. Unfortunately, mechanisms with regard to metastasis are not yet fully understood.

The establishment of human cancer cell lines/strains would be useful in the study of tumour development and progression. For several technical reasons, the success rate for establishment of carcinoma cell lines is extremely low, commonly less than 2% (Park et al, 1990; Amadori et al, 1993; Hambly et al, 1997), especially for cell lines with metastatic properties.

Krause summarized three factors affecting the success of culture growth (Krause, 1981). The highest cause of failure is a lack of adequate viable cells, which are not likely to thrive in vitro. The overgrowth of promising tumour cells by fibroblasts is another. A xenograft from an ideal animal model bearing human tumour would provide more viable cells compared with surgical samples used directly to establish cell lines. Our institute previously developed a metastatic model of human HCC in nude mice (LCI-D20) (Sun et al, 1996), which could provide ideal cancer cells for the establishment of a metastatic cell line. The establishment of MHCC97 suggested that the success rate would be higher by means of repeated combination of culture in vitro and growth in nude mice than by conventional culture methods; and the dispersed cells by mechanical isolation would be superior to tissue fragment for culture. The former may make the cells acquire the phenotype for long-term culture in vitro and retain or regain several properties of the original tumour (Tom et al, 1977), such as AFP synthesis and lung metastasis; and the latter would reduce the contamination of fibroblasts. In addition, we also found that human blood group AB serum does not seem to suit for mouse fibroblast growth.

Based on the biological behaviour, MHCC97 possesses a highly metastatic potential, and the lungs seem to be the preferential target organ for its metastasis, which is similar to the patients with HCC in clinic.

Activation of oncogenes caused by hepatitis virus integration has been well shown in the woodchuck animal model (Hsu et al, 1988; Fourel et al, 1990). The integration of HBV into genomic DNA is associated with chromosome changes in recipient cells

**Table 2** Appearing frequency of abnormal chromosomes in 22 karyotypes analysed (35th passage)

| | Extra chromosomes | | | | | | | Abnormal chromosomes (markers) | | |
|---|---|---|---|---|---|---|---|---|---|---|
| Chromosome | –Y | +2 | +3 | –4 | +5 | +6 | +7×2 | M1 | M2 | M3 |
| Frequency | 22 | 14 | 9 | 12 | 9 | 20 | 16 | 15 | 22 | 22 |
| Chromosome | +11 | +12×2 | –13 | –14 | –15 | +16 | +18 | M4 | M5 | M6 |
| Frequency | 15 | 17 | 22 | 22 | 22 | 11 | 11 | 12 | 12 | 14 |
| Chromosome | +19 | +20 | –21×2 | | | | | M7 | M8 | |
| Frequency | 9 | 12 | 22 | | | | | 15 | 17 | |

*Appearing frequency of abnormal chromosomes exceeded 50% of karyotypes analysed.

 *© 1999 Cancer Research Campaign*

**Table 3** Abnormal chromosomes for MHCC97 cell lines

| Chromosomes | Markers |
|---|---|
| 1(M1) | Inv(1)(pter→q32::q42→q41::q43→qter) |
| 1(M2) | i(1)(q) |
| 1,15(M4) | t(1;15)(1qter→p13::15q12→qter) |
| 4(M3) | der(4)(pter→q35::?) |
| 5,9 | t(5qter→q11.2::9p13→qter) |
| 7,9 | t(7;9)(7qter→q11.1::9p11→qter) |
| 13,14(8) | t(13;14)(13qter→q14::14p112→qter) |
| 11 (M10) | del(11)(:p12→qter) |
| 9,14 (M5) | t(9;14)(9qter→q11::14p11→qter) |
| 10, 14(6) | t(10;14)(10qter→p11::14q112→qter) |
| 17,?(M7) | t(17;?)(17pter→q25::?) |
| 20(M9) | del(20)(pter→q12:) |
| X | der(x;?)(xqter→xp11.1::?→xq11→xqter) |

(Rogler, 1985). PCR-amplified products of HBsAg and HBxAg indicate that HBV may have integrated into genetic DNA of cell line MHCC97, although the line has lived for a long time in vitro.

Chromosome abnormalities are the hallmark of cancer cells. Cancer is thought to be caused by a series of alterations of a limited number of specific genes, which reflects cumulative genetic alteration, including activation of oncogenes and inactivation or loss of tumour suppressor genes (Bishop, 1991; Weinberg, 1992; Kuroki et al, 1995). Multiple genetic alterations are generally suggested to be involved in the development of HCC (Sugimura, 1992), but loss of tumour suppressor genes may be a more important genetic aberration that is responsible for HCC formation (Yeh et al, 1996). Karyotypic analysis revealed that recurring and highly consistent structural and numerical alternations were present in all the cells examined, corresponding with the characteristics of malignant tumour in genetics. Large proportions of chromosomal modal (73%) and two chromosome markers presented invariable in all cells indicated that MHCC97 may be of monoclonal origin (Liao et al, 1975). Monosomy is known to be one of the mechanisms of tumour suppressor gene loss in malignant tumours (Bishop, 1991). Kuroki et al (1995) reported that at least one putative tumour suppressor gene for HCC, other than retiroblastona, exists on chromosome 13q. Some investigators have shown that allelic losses on chromosome 13q tend to occur more frequently in advanced HCC, being apparently associated with progression of the tumours (Murakami et al, 1991). The appearance of monosomies 13,14,15 in the line may have implied that some locus of chromosome contains the putative tumour suppressor genes or metastasis suppressor genes. Trisomy is also observed in HCC. The consequences of monosomy and trisomy may result in the loss of tumour suppressor genes or amplification of oncogenes by dose effects during development and progression of tumours.

Any one of several mechanisms, such as point mutation, translocation, amplification, or loss of the whole chromosome or part of a chromosome, can activate oncogenes or inactivate tumour suppressor genes and allow a cell to escape from normal control of growth (Fujimori et al, 1991). From conventional cytogenetics it has been suggested that chromosome 1 abnormalities are associated with HCC (Simon et al, 1990, 1991; Bardi et al, 1992; Chen et al, 1993; Werner et al, 1993; Yeh et al, 1994). The most consistent aberrations detected concerning chromosome 1 in HCC are polysomy 1 or deletion or translocation involving its short arm (Simon et al, 1990; Bardi et al, 1992; Chen et al, 1993; Yeh et al, 1994). Chromosome 4q abnormality was also significantly associated with HCC (Pasquinelli, 1988; Zhang et al, 1990; Yeh et al, 1996). The strong correlation of LOH at 4q with AFP elevation in HCC suggests that loss of such putative tumour suppressor genes can also remove the repression of AFP gene expression (or allow persistent AFP gene expression) (Yeh et al, 1996). The establishment of the metastatic line may aid further study of the relation between the special aberrations of chromosomes and metastasis of HCC.

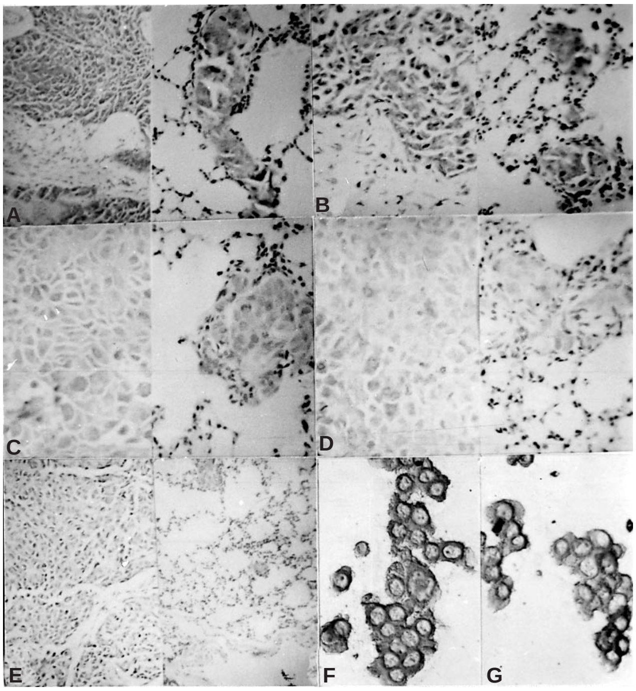

**Figure 5** Immunohistochemical micrographs. Primary antibodies used are the following: anti-c-Met (**A, F**), uPAR (**B, G**), integrin α5 (**C**), integrin β1 (**D**) and E-cadherin (**E**) antibodies respectively. **A–E** were from intrahepatic xenografts and pulmonary metastic lesions, and **G, F** from MHCC97 cells grown on coverslips. Scale bar = 50 μm (**A, B, E**) and 25 μm (**C, D, F, G**)

Invasion and metastasis are the greatest obstacles to successful tumour treatment. The metastasis is a complex and multistep process (Aznavoorian et al, 1993; Nigam et al, 1994). For tumour cells to metastasize, they must acquire the ability to detach from the primary tumour, and penetrate and exit through the wall of the vascular and/or lymphatic circulatory system, and undergo the cell proliferation and angiogenesis required for colonization in a target organ (Nigam et al, 1994). Gene products that regulate the processes of cell adhesion, ECM/BM degradation, cell motility, cell growth and angiogenesis could contribute to the metastatic potential. Many of these phenotypes have been associated with adhesive molecules, activation and secretion of proteinases, motility factor, growth factors and VEGF (Nigam et al, 1994), which could play a role in metastasis. It is important to explore the expression level of their relevant factors for understanding metastatic mechanisms.

Integrin $\alpha_5$, $\beta_1$ is identified as one of the fibronectin (FN) receptors. The loss, reduction or redistribution of α5, β1 on cell surfaces is implicated in detachment of adhering cells from ECM timely for metastasis (Giancutti and Ruoslahti, 1990; Schreiner et al, 1991; Albelda, 1993; Aznavoorian et al, 1993). E-cadherin is

© 1999 Cancer Research Campaign also an important adhesion molecule in inhibiting tumour metastasis by connecting homophilic cells (Frixen et al, 1991). Its down-regulation is implicated in metastasis of some malignant tumours (Shimoyama and Hirohashi, 1991; Miyasaka, 1995). uPA is a serine proteinase responsible for the degradation of ECM/BM and activation of hepatocyte growth factor (HGF) (Daikuhara et al, 1992; Ellis et al, 1992; Blasi, 1993). Active uPA is found predominantly at the cell surface, where it is retained by a high-affinity receptor (uPAR). HGF is a pleiotropic effector of cells expressing its receptor c-Met. HGF/Met signalling by stimulating growth and migration of malignant cells enhances the tumorigenicity and metastasis of cancer cells in vivo (Jeffers et al, 1996; Matsumoto and Nukamura, 1996; Weidner et al, 1990). It can also elevate the protein levels of both uPA and its receptor on cancer cells (Jeffers et al, 1996). In addition, HGF/Met can also stimulate neo-angiogenesis in vivo (Bussolino et al, 1992; Grant et al, 1993). VEGF expressed in tumours is identified as responsible for the formation of tumour vessels that not only allow for progression of the primary tumours, but also facilitate cancer cells to access to the tumour vessels (Fildler and Ellis, 1994; Koch et al, 1994).

The positive expressions of integrin $\alpha5$, $\beta1$ indicated that the cell line MHCC97 has acquired the ability to adhere to ECM/BM, but its negative expression in metastaic lesions facilitates MHCC97 cells to detach in a timely manner from ECM/BM for metastasis. One of HGF effects is just contrary to one of E-cadherin. The up-modulation of HGF/c-Met and down-modulation of E-cadherin may result in the dispersion/scattering of MHCC97 cells, which may be favourable for metastasis. High expression of uPAR would confer uPA on high active state, which promotes the degradation of ECM/BM. It is not surprising that high expression of VEGF is indispensable for MHCC97 cell metastasis. The metastatic suppressor gene nm23 has been shown to promote the growth of some tumours by cell proliferation following further studies besides inhibiting metastasis (Igawa et al, 1994).

Our data indicated that the phenotype of cell line MHCC97 corresponds to the characteristics of human HCC and retain some properties of original tumour, including AFP synthesis and metastatic potential. The findings of factors associated with metastasis further proved that cell line MHCC97 possesses the prerequisites for metastasis. Integration of HBV into MHCC97 cells suggests that HBV may participate in initiation and development of HCC.

### ACKNOWLEDGEMENT

This work was partly supported by the China Medical Board of New York, Grant No. 93-583 ‘Primary Liver Cancer’. This work was supported by ‘State Key Basic Research Program Grant G1998051211’.

### REFERENCES

Albelda SM (1993) Role of integrins and other cell adhesion molecules in tumour progression and metastasis. *Lab Invest* **68**: 4–17

Alexander JJ, Bey EM and Lecates G (1976) Establishment of a continuously growing cell line derived from primary carcinoma of the liver. *J South Afr Med* **50**: 2124–2128

Amadori D, Bertoni L, Flamigni A, Savini S, Giovanni CD, Casanova S, de Paola F, Amadori A, Giulotto E and Zoli W (1993) Establishment and characterization of a new cell line from primary human breast carcinoma. *Breast Cancer Res Treat* **28**: 251–260

Anderson BB, Ukah F, Tette A, Villaflor SG, Koh D and Seton P (1992) Primary tumors of the liver. *J Natl Med Assoc* **84**: 129–135

Aznavoorian S, Murphy AN, Stetler-Stevenson WG and Liotta LA (1993) Molecular aspects of tumor cell invasion and metastasis. *Cancer* **71**: 1368–1383

Bardi G, Johanssan B, Pandis N, Mandahl N, Andren-Sandberg A, Hugerstrand I and Mitelman F (1992) Cytogenetic findings in three primary hepatocellular carcinoma. *Cancer Genet Cytogenet* **58**: 191–195

Bishop JM (1991) Molecular themes in oncogenesis. *Cell* **64**: 235–248

Blasi F (1993) Urokinase and urokinase receptor: a paracrine/autocrine system regulating migration and invasiveness. *Bioassays* **15**: 105–111

Bussolino F, Di Renzo MF, Ziche M, Bocchietto E, Olivero M, Naldimi L, Gaudino G, Tamagnone L, Coffer A and Comoglio PM (1992) HGF is a potent angiogenic factor which stimulates endothelial cell motility and growth. *J Cell Biol* **119**: 629

Chen HL, Chiu TS, Chen PJ and Chen DS (1993) Cytogenetic studies on human liver cancer cell lines. *Cancer Genet Cytogenet* **65**: 161–166

Chen RM (1963) The establishment of a strain of human liver carcinoma in vitro and some preliminary observations. *Chin Med J* **82**: 228–237

Daikuhara H, Tsubouchi F, Blasi F and Comoglis PM (1992) Extracellular proteolytic cleavage by urokinase is required for activation of HGF/scatter factor. *EMBO J* **11**: 4825–4833

Dong RC, Zhou RH, Lu FD and Tao WZ (1980) Establishment of a human hepatocarcinoma cell line SMMC-7721 and initial observations on its biologic characteristics. *Acad J Second Millitary Med Univ* **1**: 5–7

Ellis VC, Pyke C, Eriksen J, Solberg H and Dano K (1992) The urokinase receptor: involvement in cell surface proteolysis and cancer invasion. *Ann N Y Acad Sci* **667**: 13–31

Fildler I and Ellis L (1994) The implications of angiogenesis for the biology and therapy of cancer metastasis. *Cell* **79**: 185–188

Fourel G, Trepo C, Baugueleret L, Hengliein B, Ponzetto A, Tiollais P and Buendia MA (1990) Frequent activation of N-myc genes by hepadnavirus insertion in woodchuck liver tumors. *Nature* **347**: 274–280

Frixen UH, Behrans HJ and Sacks K (1991) E-cadherin-mediated cell–cell adhesion prevents invasiveness of human carcinoma cells. *J Cell Biol* **113**: 173–185

Fujimori M and 12 others (1991) Allelotype study of primary hepatocellular carcinoma. *Cancer Res* **51**: 89–93

Giancutti FG and Ruoslahti E (1990) Elevated level of $\alpha5\beta1$ fibronectin receptor suppress the transformed phenotype in CHO cells. *Cell* **60**: 849–859

Giard DJ, Aronson SA, Todaro GJ, Arnstein P, Kersey JH, Dosik H and Parks WP (1973) In vitro cultivation of human tumors: establishment of cell lines derived from a series of solid tumors. *J Natl Cancer Inst* **51**: 1417–1423

Grant DS, Kleinman HK, Goldgerg ID, Bhargava MM, Nichaloff BJ, Kinsella JL, Polverini P and Rosen EM (1993) Scatter factor induces blood vessel formation in vivo. *Proc Natl Acad Sci USA* **90**: 1937–1941

Hambly RJ, Double JA, Thompson MJ and Bibby MC (1997) Establishment and characterization of new cell lines from human breast tumors initially established as tumor xenografts in NMRI nude mice. *Breast Cancer Res Treat* **43**: 247–258

He L, Isselbacher KJ, Wands JR, Goodman HM, Shih C and Quaroni A (1984) Establishment of characterization of a new HCC cell line. *In Vitro* **20**: 493–504

Hsu TY, Moroy T, Etiemble J, Louise A, Trepo C, Tiollais P and Buedia MA (1988) Activation of c-myc by woodchuck hepatitis virus insertion in hepatocellular carcinoma. *Cell* **55**: 627–635

Igawa M, Rukstalis DM, Tanabe T and Chodak GW (1994) High level of nm23 expression are related to cell proliferation in human prostate cancer. *Cancer Res* **54**: 1313–1318

Jeffers M, Rong S and Van Woude GF (1996) Enhanced tumorigenicity and invasion-metastasis by HGF/SF-Met signaling in human cells consistent with induction of the urokinase proteolysis network. *Mol Cell Biol* **16**: 115–1125

Koch AE, Harlow LA, Haines GK, Amento EP, Unemori EN, Wong WL, Pope RM and Ferrara N (1994) Vascular endothelial growth factor. A cytokine modulating endothelial function in rheumatoid arthritis. *J Immunol* **152**: 4149–4156

Krause CJ and Beekell MA (1981) Tumorigenic keratinocyte lines requiring anchorage and fibroblast support cultured for human squamous cell carcinomas. *Cancer Res* **41**: 1657–1663

Kuroki T, Fujiwara Y, Nakamori S, Imaoka S, Kanematsu T and Nakamura Y (1995) Evidence for the presence of two tumor-suppressor genes for hepatocellular carcinoma on chromosome 13q. *Br J Cancer* **72**: 383–385

Liao SK, Dent PB and McCulloch PB (1975) Characterization of human malignant melanoma cell line. I. Morphology and growth characteristics in culture. *J Natl Cancer Inst* **54**: 1037–1044

Matsumoto K and Nakamura T (1996) Emerging multiprotent aspects of hepatocyte growth factor. *J Biochem Tokyo* **119**: 591–600

Meltzer P, Leibovita A, Dalton W, Villar H, Kute T, Davis J, Nagle R and Trent J (1991) Establishment of two new lines derived from human breast carcinomas with HER-2/ueu amplification. *Br J Cancer* **63**: 727–735

 © 1999 Cancer Research Campaign

Miyasaka M (1995) Cancer metastasis and adhesion molecules. *Clin Orthop Relat Res* **312**: 10–18

Murakami Y, Hayashi K, Hirohashi S and Sekiya T (1991) Aberration of the tumor suppressor p53 and retinoblastoma genes in human hepatocellular carcinoma. *Cancer Res* **51**: 5520–5525

Nigam AK, Pignatelli M and Boulos PBC (1994) Current concepts in metastasis. *Gut* **35**: 996–1000

Nishida N, Fukuda Y, Kuroki H, Sadamoto T, Isowa G, Honda K, Yamaoka Y, Ikenaga M, Imura H and Ishizaki K (1992) Accumulation of allelic loss on arms of chromosomes 13q, 16q and 17p in the advanced stages of human hepatocellular carcinoma. *Int J Cancer* **51**: 862–868

Okuda K (1992) Hepatocellular carcinoma: recent progress. *Hepatology* **15**: 948–963

Park JG and 12 others (1990) Characteristics of cell lines established from human gastric carcinoma. *Cancer Res* **50**: 2773–2780

Parkin DM, Pisani P and Ferlay J (1993) Estimates of worldwide incidence of eighteen major cancers in 1985. *Int J Cancer* **54**: 594–606

Pasquinelli C, Garreau F, Bougueleret L, Cariani E, Grzeschik KH, Thiere V, Croissant O, Hadchauel M, Tiollais P and Brechot C (1988) Rearrangement of a common cellular DNA domain on chromosome 4 in human primary liver tumour. *J Virol* **62**: 629

Rogler CE, Sherman M, Su CY, Shafritz DA, Summers J, Shows TB, Henderson A and Kew M (1985) Deletion in chromosome 11p associated with a hepatitis B integration site in hepatocellular carcinoma. *Science* **230**: 319–322

Schreiner C, Fisher M, Hussein S and Juliano RL (1991) Increased tumorigenicity of fibronectin receptor deficient Chinese hamster ovary cell variants. *Cancer Res* **51**: 1738–1740

Seabright MA (1971) A rapid banding technique for human chromosomes. *Lancet* **ii**: 971–972

Shimoyama Y and Hirohashi S (1991) Cadherin intercellular adhesion molecule in hepatocellular carcinoma: loss of E-cadherin expression in an undifferentiated carcinoma. *Cancer Letts* **57**: 131–135

Simon D, Munou SJ, Maddrey WC and Knowles BB (1990) Chromosomal rearrangements in a primary hepatocellular carcinoma. *Cancer Genet Cytogenet* **45**: 255–260

Simon D, Knowles BB and Weith A (1991) Abnormalities of chromosome 1 and loss of heterozyosity on 1p in primary hepatomas. *Oncogene* **6**: 765–770

Sugimura T (1992) Multistep carcinogenesis: a 1992 prospective. *Science* **258**: 603–607

Sun FX, Tang ZY, Liu KD, Ye SL, Xue Q, Gao DM and Ma ZC (1996) Establishment of a metastatic model of human hepatocellular carcinoma in nude mice via orthotopic implantation of histologically intact tissue. *Int J Cancer* **66**: 239–243

Tom BH, Rutzky LP, Oyasu R, Tomita JT, Goldberg DM and Kahan BD (1977) Human colon adenocarcinoma cell. II. Tumorigenic and organoid expression in vivo and in vitro. *J Natl Cancer Inst* **58**: 1507–1512

Weidner KM, Behrens J, Vandekerckhove J and Birdhmeier W (1990) Scatter factor: molecular characteristics and effect on the invasiveness of epithelial cells. *J Cell Biol* **111**: 2097–2108

Weinberg RA (1992) The integration of molecular genetics into cancer management. *Cancer* **70**: 1653–1658

Werner M, Nolte M, Klempanauer J and Georgii A (1993) Chromosome 1 abnormalities in hepatocellular carcinoma. *Cancer Genet Cytogenet* **64**: 130–135

Yeh SH, Chen PJ, Chen HL, Lai MY, Wang CC and Chen DS (1994) Frequent genetic alternations at the distal region of chromosome 1p in human hepatocellular carcinoma. *Cancer Res* **54**: 4188–4192

Yeh SH, Chen PJ, Lai MY and Chen DS (1996) Allelic loss on chromosome 4q and 16q in hepatocellular carcinoma: association with elevated α-fetoprotein production. *Gastroenterology* **110**: 184–192

Zhang W, Hirohashi S, Tsuda H, Shimosato Y, Yokota J, Teradam M and Sugimura T (1990) Frequent loss of heterozygosity on chromosomes 16 and 4 in human hepatocellular carcinoma. *Jpn J Cancer Res* **81**: 108–111

*© 1999 Cancer Research Campaign*
